# How chronotype, sleep-wake cycle, subjective time experience influence retrospective, and prospective memory functioning

**DOI:** 10.3389/fcogn.2025.1683207

**Published:** 2025-10-17

**Authors:** Marco Fabbri, Monica Martoni

**Affiliations:** ^1^Department of Psychology Renzo Canestrari, University of Bologna, Bologna, Italy; ^2^Department of Medical and Surgical Sciences, University of Bologna, Bologna, Italy

**Keywords:** morningness-eveningness preference, sleep-wake cycle, subjective passage of time, retrospective memory, prospective memory, time pressure, time expansion/boredom, daytime sleepiness

## Abstract

**Background:**

The sleep-wake cycle and chronotype are key contributors to memory consolidation. Emerging evidence also highlights their role in shaping the subjective experience of time, which, in turn, can influence cognitive performance, particularly memory. This study investigated how sleep-wake quality, circadian typology, and subjective time perception relate to failures in retrospective and prospective memory.

**Method:**

A total of 666 participants (73% female; M = 37.83 years, SD = 14.65) completed the reduced Morningness-Eveningness Questionnaire (rMEQ), Mini-Sleep Questionnaire (MSQ), Prospective and Retrospective Memory Questionnaire (PRMQ), Subjective Time Questionnaire (STQ), and *ad hoc* questions about sleep habits during weekdays and weekends.

**Results:**

Results indicated that evening-types reported later sleep and wake times, more pronounced sleep-wake disturbances, greater memory lapses, and stronger experiences of time expansion/boredom. Correlational analyses showed that both time pressure and time expansion/boredom were negatively associated with PRMQ scores, leading to increased memory errors. The regression models showed that memory performance was generally predicted by wake factor, time pressure and time expansion/boredom. Then, mediation models revealed that eveningness was associated with greater wake problems, which were positively related to altered time perception (i.e., time pressure and time expansion/boredom), which in turn predicted more frequent retrospective and prospective memory failures.

**Discussion:**

These findings suggest that circadian typology, wake-related disturbances, and subjective time perception interactively contribute to memory performance, and propose a model linking biological rhythms and temporal experience to memory functioning, suggesting a possible influence of attention and arousal.

## 1 Introduction

Memory refers to the capacity to encode, store, and retrieve information derived from experience (Tulving and Craik, 2000). It is commonly conceptualized as a multifaceted construct comprising distinct component processes, which may be recruited in varying combinations depending on situational demands and are typically assessed through specialized paradigms (Squire, 2004). One well-established distinction within the memory domain is that between retrospective memory, which concerns the recall of past events, and prospective memory, which involves remembering to execute intended actions in the future (Maylor et al., 2002; Smith et al., 2000). Prospective memory itself can be classified as either event-based, where actions are triggered by external cues, or time-based, in which actions are executed at a specific time or following a temporal delay (Kvavilashvili, 1987). According to the dynamic multiprocess framework, both event-based and time-based prospective memory tasks can rely on either strategic, attentionally mediated monitoring or on spontaneous retrieval, whereby the appropriate temporal or contextual cue automatically activates the stored intention (Einstein et al., 2005; Scullin et al., 2013). Irrespective of task type, prospective memory entails two functionally distinct components (Kliegel et al., 2007): the prospective component (remembering that an action must be performed) and the retrospective component (remembering the content of the intended action). Given their crucial role in daily functioning, such as remembering medication schedules or navigating familiar routes, it is essential to examine which factors influence both prospective and retrospective memory performance. In this regard, the Prospective and Retrospective Memory Questionnaire (PRMQ) has been developed and validated to assess self-reported memory failures in everyday life, distinguishing between prospective and retrospective components (Smith et al., 2000; Crawford et al., 2003). The PRMQ comprises 16 items, equally divided across memory types, and further differentiates between self-initiated and environmentally cued failures, as well as between short- and long-term contexts. Thus, the PRMQ is a reliable tool for assessing several components of episodic memories.

Human physiology, behavior, and cognition exhibit circadian rhythmicity, entrained by the environmental light–dark cycle. The two-process model of sleep regulation (Borbély et al., 2016) posits that sleep–wake patterns are governed by the interaction between a homeostatic process (S), which accumulates sleep pressure across wakefulness, and a circadian process (C), which generates near-24-h endogenous oscillations (Borbély and Achermann, 1999). These circadian rhythms are regulated by the suprachiasmatic nuclei (SCN) in the hypothalamus (also known as the central biological clock; Taillard et al., 2021), which synchronizes endogenous rhythms to external zeitgebers such as natural and artificial light exposure, as well as social schedules including work and school obligations (Kelley et al., 2018; Taillard et al., 2021). Misalignment between biological and social time, commonly termed social jetlag, emerges when imposed schedules conflict with endogenous circadian preferences (Wittmann et al., 2006; Roenneberg et al., 2019). Such circadian misalignment has been shown to detrimentally affect memory encoding and retrieval (Cho, 2001; Eckel-Mahan et al., 2008; Smies et al., 2022) and is frequently observed in neuropsychiatric conditions characterized by memory dysfunction, including Alzheimer's disease, major depression, and post-traumatic stress disorder (Albrecht and Stork, 2017; Homolak et al., 2018; Vadnie et al., 2021). Thus, the timing of biological clock seems to be relevant to memory processes. These considerations find further support in reviewing that learning and memory show circadian modulation, both in terms of memories activation, and in terms of memories formation (Smarr et al., 2014). Specifically, it has been found that SCN might play a role in encoding time-of-day information, whereas the hippocampus encodes time-of-day into new memories and exhibits time-of-day modulation in encoding and recall efficiency. Thus, a misalignment between SCN and hippocampus leads to recall errors (Smarr et al., 2014).

Circadian typology, or chronotype, reflects stable interindividual differences in preferred timing of daily activities and sleep-wake behavior (Adan et al., 2012). Chronotypes are generally distributed along a continuum from morningness to eveningness, with approximately 40% of individuals classified as extreme types and the remaining as intermediate-types. Morning-types tend to wake and function optimally earlier in the day, whereas evening-types exhibit delayed sleep–wake phases and peak cognitive performance in the evening hours (Chauhan et al., 2025; May et al., 2023; Schmidt et al., 2007; Taillard et al., 2021; Wiłkość-Dȩbczyǹska and Liberacka-Dwojak, 2023). Intermediate-types show neither strong morningness nor strong eveningness preferences and experience a peak in body temperature that is somewhere between that of morning-types and evening-types (Roenneberg et al., 2007). In the literature, little is known about intermediate-types and whether their performance varies over the day (Adan et al., 2012; Chauhan et al., 2025; May et al., 2023). These behavioral profiles are thought to result from differential interaction between homeostatic and circadian processes (Mongrain et al., 2006). For instance, morning-types show a more rapid buildup of sleep pressure across the day, whereas evening-types exhibit a delayed circadian phase and a slower accumulation of homeostatic sleep drive. Consequently, evening-types are more likely to experience social jetlag, accumulating sleep debt during weekdays and compensating with longer sleep durations on weekends (Taillard et al., 1999; Wittmann et al., 2006). The phase-delayed profile of evening-types has been associated with greater variability in cognitive performance, particularly under conditions of temporal misalignment.

Empirical findings suggest that chronotype modulates cognitive performance, including memory. Petros et al. (1990) reported superior prose recall in evening-types relative to morning-types, while other studies have shown enhanced cognitive flexibility and semantic processing in morning-types under specific conditions (Palmero et al., 2024). Fabbri et al. (2013) demonstrated that both extreme chronotypes exhibited faster classification of semantically dominant items in a categorization task, although interference effects during automatic retrieval were more pronounced in evening-types. Regarding prospective memory, Rothen and Meier (2017) found that optimal time-of-day testing was critical for performance in both chronotypes (but see Barner et al., 2019 for opposite results). The effect of chronotypes on memory could be also observed indirectly due to the differences between morning- and evening-types in sleep quality and circadian alignment, which have been repeatedly shown to affect cognitive domains, including memory consolidation, attention, and executive control (McGowan et al., 2020; Taillard et al., 2021). To best of our knowledge, May and Hasher (2017) showed that older intermediate-type individuals had better performance on inhibitory processing, executive function (with better performance than evening-types; Hicks et al., 2023), long-term memory, and forgetting, at midday (i.e., their optimal time-of-day) generally. By contrast, younger intermediate-types did not report variations of performance over the day, suggesting cognitive flexibility over the day in cognitive performance (May and Hasher, 2017).

Sleep has a well-documented role in the consolidation of both retrospective (e.g., Chambers, 2017; Diekelmann and Born, 2010; Girardeau and Lopes-Dos-Santos, 2021; Klinzing et al., 2019; Rasch and Born, 2013) and prospective memories (Barner et al., 2017; Diekelmann et al., 2013; Esposito et al., 2015; Fabbri et al., 2014, 2015; Leong et al., 2019; Occhionero et al., 2020; Scullin and McDaniel, 2010; Tonetti et al., 2020), with evidence drawn from experimental and observational paradigms. The synaptic homeostasis hypothesis (SHY; de Vivo et al., 2017) proposes that sleep supports homeostatic regulation by downscaling synaptic strength, thereby preventing saturation and enabling subsequent memory encoding. According to SHY, global synaptic weights increase during wakefulness and decrease during sleep, with cortical slow-wave activity indexing overall synaptic strength (Vyazovskiy et al., 2009). Slow oscillations rise following prolonged wakefulness and decline across successive sleep episodes, consistent with synaptic downscaling. In parallel, hippocampal neurons show progressive increases in firing rates during wakefulness and state-dependent modulation during sleep, characterized by enhanced activity in NREM and suppression in REM (Grosmark et al., 2012; Miyawaki and Diba, 2016). These dynamics indicate that sleep-dependent homeostatic processes play a critical role in memory consolidation. Indeed, sleep deprivation impairs hippocampal-dependent memory consolidation, increases false memories, disrupts contextual binding, and reduces attentional control (Ellenbogen et al., 2009; Feld et al., 2016; Fenn et al., 2009; Kim et al., 2022; Krause et al., 2017; van der Helm et al., 2011). The large-scale disruption of circadian timing during the COVID-19 pandemic (Blume et al., 2020), due to lifestyle and schedule changes, further supports the association between irregular sleep–wake patterns and subjective memory difficulties (Fabbri, 2023; Fiorenzato et al., 2021), indicating that the quality and regularity of sleep-wake cycle play a role for memory performance. For example, Fabbri et al. (2014, 2015) have found that good sleepers performed a prospective memory task better than bad sleepers, indicating the importance of quality of sleep.

In addition to circadian regulation, temporal cognition is central to a range of motor and cognitive functions, including movement timing and processing speed (Buhusi and Meck, 2005; Maniadakis and Trahanias, 2014; Pouthas and Perbal, 2004). While the impact of memory and attention on time perception has been extensively examined (Block et al., 2010; Block and Gruber, 2014; Matthews and Meck, 2016), few studies have addressed whether subjective time perception, particularly passage-of-time judgments (POTJ), influences memory processes. The subjective POT refers to the perception that time is moving faster or slower than clock time (Droit-Volet and Martinelli, 2023; Martinelli and Droit-Volet, 2022; Wittmann et al., 2015; Wearden, 2015). Episodic memory entails integration of “what,” “where,” and “when” components into a coherent representation (Marshall et al., 2013), and, thus, “errors” in subjective time perception could determine failures in remembering. Moreover, memory and time processes share neural substrates including the prefrontal cortex, anterior cingulate, supplementary motor area, parietal cortex, hippocampus, cerebellum, and basal ganglia (Addis et al., 2009; Leon and Shadlen, 2003; Meck et al., 2008; Rolls, 2022). These structures are implicated not only in interval timing but also in subjective time flow, or POTJ (Coslett et al., 2009; Miloyan et al., 2019; Muller and Nobre, 2014; Nyberg et al., 2010). Consequently, the assessment of subjective awareness of the POT could give insight into the possible failures of retrospective and prospective memories.

The present study examined whether circadian typology, sleep–wake quality, sleep timing regularity, and subjective POTJ independently or interactively predicted failures in prospective and retrospective memory. Previous findings have shown that morning-types tend to overestimate time intervals and report a faster subjective time flow, whereas evening-types show the opposite pattern (Beracci et al., 2022; Esposito et al., 2007). Such differences may reflect chronotype-specific temporal cognition, potentially impacting long-term memory processes. Fabbri (2023) reported that increased attentional focus on the present-moment was associated with greater memory failures and reduced subjective time expansion. Chronotype may therefore modulate memory indirectly through effects on temporal awareness, sleep quality, and sleep regularity. For instance, greater perceived time slowing or boredom (Zakay, 2014) has been shown to delay bedtime and compromise sleep continuity (Droit-Volet et al., 2020, 2021; Martinelli et al., 2021; Teoh and Wong, 2023; Wittmann, 2020), leading to cumulative cognitive costs. Experimental studies have further demonstrated that sleep deprivation alters prospective and retrospective time estimation, typically shortening perceived intervals in prospective timing and lengthening them in retrospective conditions (Casini et al., 2013; Sen et al., 2023; Soshi et al., 2010).

Taken together, these findings support a conceptual model Firstly, the current study aimed to test, for the first time, whether (and how) circadian typology, sleep-wake quality, sleep timing, and subjective awareness of time flow could influence prospective and retrospective memory functioning. Secondly, the present study aimed to test a possible model whereby chronotype is associated with sleep–wake parameters and subjective POTJ, which in turn influence self-reported memory performance. Specifically, it was predicted that eveningness, sleep-wake disorders, and altered slow passage of time (time pression or expansion/boredom) could contribute to predict more memory failures. POTJ was operationalized using subjective estimates of time passage over extended intervals (e.g., “*How fast did last week pass?*”) and through assessments of time-related phenomenology such as time pressure and boredom (Droit-Volet and Martinelli, 2023; Wittmann and Lehnhoff, 2005).

## 2 Method

### 2.1 Participants

A total of 666 individuals (mean age = 37.83 years, SD = 14.65 years; range = 18–73 years) participated in this cross-sectional study. The sample comprised 73% females and 27% males. Participation was voluntary, anonymous, and unpaid, with the option to withdraw at any time. Recruitment occurred through psychology, medicine, and nursing courses, as well as via snowball sampling. The study was approved by the Ethics Committee of the Department of Psychology at the University of Campania Luigi Vanvitelli (Protocol No. 04/2016, approved February 23, 2016), where the corresponding author was affiliated for the period 2012–2024. All participants provided written informed consent in accordance with the 1964 Helsinki Declaration.

### 2.2 Materials

#### 2.2.1 Morningness-eveningness questionnaire—reduced version (rMEQ)

The Italian version of the rMEQ (Adan and Almirall, 1991; Natale, 1999) was used to assess chronotype. This five-item questionnaire derives from the original 19-item MEQ (Horne and Östberg, 1976) and includes questions on preferred bed/wake times, peak alertness, post-awakening tiredness, and self-assessed chronotype. Scores range from 4 to 25: morning-type (>18), evening-type (<11), and intermediate-type (11–18). In the sample, 9.9% were evening-types (*n* = 66), 66.4% intermediate-types (*n* = 442), and 23.7% morning-types (*n* = 158).

#### 2.2.2 Mini-sleep questionnaire (MSQ)

The Italian MSQ (Natale et al., 2014) assessed the frequency of 10 sleep/wake-related behaviors over the past 2 weeks using a 7-point Likert scale (1 = never, 7 = always). Total scores indicate overall sleep-wake problems. Two subscales were derived: MSQ Sleep (5 items) and MSQ Wake (4 items) factors, with item 6 (snoring) excluded from Sleep factor. Higher scores on each subscale reflect greater dysfunction. Internal consistency was acceptable (Total α = 0.85; MSQ Sleep α = 0.81; MSQ Wake α = 0.77). Based on Natale et al.'s cut-offs, 45.8% had sleep problems (>16), and 47.7% showed excessive daytime sleepiness (>14). Categorization yielded 38.6% with no issues, 29.3% with either sleep or wake issues, and 32.1% with both.

#### 2.2.3 Sleep-Wake Habits (SWH)

Four *ad hoc* questions (Fabbri, 2023) assessed typical sleep-wake behaviors, such as the normal time participant go to bed (bedtime or BT) and wake time (WT) in the morning, on workdays (WBT, WWT) and free days (FBT, FWT), from which Time in Bed (TIB) and Social Jetlag (SJL) were calculated. The TIB was calculated as the number of hours and minutes in bed from the moment of bedtime to that of wake time. Midpoint of Sleep (MPoS, as the exact halfway point of TIB) was computed for each condition to derive SJL as the absolute difference between free-day and workday MPoS (Wittmann et al., 2006).

#### 2.2.4 Time awareness and subjective time questionnaire (TASTQ)

The Italian version of the TASTQ (Torboli et al., 2023; Wittmann and Lehnhoff, 2005) measures subjective time perception and awareness. Present Experience (PE) and Past Time (PT) were assessed on a scale from −2 (very slow) to +2 (very fast). PE included two items on current and expected passage of time; PT included four items on the perceived speed of past intervals (e.g., last week, month, year, decade). Participants also rated Time Pressure (TP) and Time Expansion/Boredom (TE/B) via two five-item subscales (1 = strongly disagree, 5 = strongly agree).

#### 2.2.5 Prospective and retrospective memory questionnaire (PRMQ)

The PRMQ (Italian version: Crawford et al., 2003; Fabbri, 2023; Fiorenzato et al., 2021; Smith et al., 2000) assesses memory failures in daily life across 16 items (5-point scale: 1 = never, 5 = very often). It includes 8 items each for prospective (Pro) and retrospective (Retro) memory, further classified by short- vs. long-term, and self- vs. environment-cued memory. Raw Pro and Retro scores were converted into T-scores using Crawford's online tool (www.psyc.abdn.ac.uk/homedir/jcrawford/ormq.htm). Higher scores indicate better memory. Cronbach's alpha was 0.89 (Pro = 0.84; Retro = 0.77).

### 2.3 Procedure

Participants completed paper-and-pencil questionnaires in a fixed order after providing informed consent. Demographic data were collected first, followed by the rMEQ, sleep-wake habits, PRMQ, MSQ, and TASTQ. A description of the study was presented during university courses, and all volunteers were invited to fill in all questionnaires. Sessions took place individually or in small groups in class, lasting approximately 20 min. A debriefing was provided at the end, and participants were encouraged to propose the survey to other individuals. All participants were trained on how to introduce the study, for instance to their parents or friends. When potential future participants expressed interest in the survey, the researchers were notified to arrange questionnaire administration. No specific exclusion criteria were applied, apart from the requirement that participants be proficient in Italian and of legal age, as both were necessary for completing the self-report measures and providing informed consent.

### 2.4 Data analysis

Analyses were performed using SPSS. Both categorical and dimensional approaches were used. A MANCOVA was conducted with Chronotype as the between-subjects factor and gender and age as covariates. Dependent variables included MSQ scores, sleep-wake habits, subjective time measures (PE, PT, TP, TE/B), and PRMQ scores. Then, partial correlations between variables were computed controlling for gender and age. Subsequentially, three stepwise linear regressions were conducted using rMEQ, MSQ (Sleep and Wake), sleep-wake habits, PE, PT, TP, and TE/B to predict PRMQ, Pro, and Retro scores. Finally, three mediation models were tested (Hayes' PROCESS macro, Model 6; Hayes, 2017), assessing whether rMEQ predicted memory via MSQ Wake and time perception (TP, TE/B), controlling for gender and age. Bootstrapping (5,000 samples; 95% CI) was used to test indirect effects (Wen and Ye, 2014). A conservative alpha level of 0.01 was adopted due to multiple comparisons (Beracci et al., 2022; Fabbri, 2023).

## 3 Results

First of all, we tested whether and how demographic characteristics were associated with each other or other variables. No gender differences in age were found, *t*_(664)_ = −0.66, *p* = 0.51, *Cohen's d* = 0.06 (males: 37.22 ± 15.62 years; females: 38.06 ± 14.29 years). The Table 1 summarizes the associations between demographic information and the variables considered in this study. According to the correlation pattern, gender and age were included as covariates in the subsequent analyses.

**Table 1:** The *r* values of correlation coefficients between demographic information and other variables.

	**Sleep factors**	**Wake factors**	**WBT**	**WWT**	**WTIB**	**WMPoS**	**FBT**	**FWT**	**FTIB**	**FMPoS**	**SJL**	**PE**	**PT**	**TP**	**TE/B**	**Total PRMQ score**	**PRO score**	**RETRO score**
Gender	**+0.19** ^*^	**+0.24** ^*^	**−0.11** ^*^	−0.05	+0.05	−0.09	**−0.10** ^*^	−0.05	+0.06	−0.08	−0.02	+0.03	+0.08	**+0.16** ^*^	−0.02	**−0.16** ^*^	**−0.19** ^*^	−0.10°
Age	+0.07	**−0.14** ^*^	**−0.24** ^*^	**−0.36** ^*^	**−0.16** ^*^	**−0.35** ^*^	**−0.53** ^*^	**−0.56** ^*^	−0.05	**−0.60** ^*^	**−0.42** ^*^	+0.01	+0.03	−0.06	−0.08	−0.07	**−0.12** ^*^	−0.004
Mean (SD)	16.45 (5.99)	14.43 (4.79)	23:38 (01:14)	07:20 (01:25)	07:42 (01:22)	03:29 (01:08)	24:58 (01:40)	09:05 (01:41)	08:06 (01:25)	05:01 (01:31)	01:32 (01:13)	0.40 (0.68)	0.80 (0.70)	3.49 (0.73)	2.41 (0.83)	50.75 (10.89)	49.88 (11.15)	49.98 (10.13)

In bold are the significant correlations. Finally, the mean (and relative SD) of total sample for each variable is reported in the last row of the table.

WBT, Working Bed Time; WWT, Working Wake Time; WTIB, Working Time In Bed; WMPoS, Working Mid-Point of Sleep; FBT, Free Bed Time; FWT, Free Wake Time; FTIB, Free Time In Bed; FMPoS, Free Mid-Point of Sleep; SJL, Social JetLag; PE, Present Experience; PT, Past Time; TP, Time Pressure; TE/B, Time Expansion/Boredom; Total PRMQ score, Total Prospective-Retrospective Memory Questionnaire score; PRO score, Prospective score; RETRO score, Retrospective score. In addition, ^*^p < 0.01 and °p = 0.01.

The MANCOVA, with Chronotype as a between-subjects factor and gender and age as covariates, revealed a significant main effect of chronotype on several variables: bedtimes (BT) and wake times (WT) on both workdays and free days, corresponding midpoints of sleep (MPoS), all PRMQ scores, the MSQ sleep and wake factors, and the Time Expansion/Boredom (TE/B) subscale of the TASTQ (see Table 2 for full results). *Post-hoc* comparisons for the MSQ sleep factor indicated that evening-types reported significantly greater sleep problems than both intermediate- and morning-types (*p* = 0.0001 for both comparisons). Regarding the MSQ wake factor, scores decreased progressively across chronotypes from evening- to intermediate- to morning-types, reflecting a linear relationship with rMEQ scores (*p* = 0.0001 for all comparisons). As expected, evening-types reported significantly later BT and WT on both weekdays and free days compared to intermediate-types, who in turn reported later times than morning-types (*p* = 0.0001 for all comparisons, except *p* = 0.003 between intermediate- and morning-types). Correspondingly, MPoS values followed the same gradient, with evening-types showing later midpoints than intermediate-types, who again reported later midpoints than morning-types (*p* = 0.0001 for all comparisons). For subjective time perception, *post-hoc* analyses revealed that evening-types experienced significantly greater TE/B than morning-types (*p* = 0.003), suggesting a higher sense of temporal under-stimulation or boredom. Finally, morning-types scored significantly higher than both intermediate- and evening-types on all PRMQ scales, indicating better self-reported memory performance across prospective and retrospective domains (*p* = 0.0001 for all comparisons)

**Table 2:** The means (and their relative SDs) of each variable for evening-, intermediate-, and morning-types are reported.

**Variable**	**Chronotypes**	**M**	**SD**	***F*_(2, 661)_**	***p***	**η^2^*_*p*_***
Sleep Factor	Evening-types	18.95	5.90	**11.34**	**0.0001**	**0.03**
	Intermediate-types	16.46	6.05			
	Morning-types	15.37	5.55			
Wake Factor	Evening-types	17.89	4.54	**31.01**	**0.0001**	**0.09**
	Intermediate-types	14.63	4.64			
	Morning-types	12.41	4.36			
WBT	Evening-types	24:37	01:19	**43.33**	**0.0001**	**0.12**
	Intermediate-types	23:44	01:09			
	Morning-types	22:56	01:02			
WWT	Evening-types	08:11	01:42	**17.19**	**0.0001**	**0.05**
	Intermediate-types	07:26	01:23			
	Morning-types	06:43	01:08			
WTIB	Evening-types	07:34	01:31	2.55	0.08	0.01
	Intermediate-types	07:42	01:21			
	Morning-types	07:46	01:21			
WMPoS	Evening-types	04:25	01:19	**39.82**	**0.0001**	**0.11**
	Intermediate-types	03:35	01:04			
	Morning-types	02:50	00:52			
FBT	Evening-types	02:13	01:33	**33.52**	**0.0001**	**0.09**
	Intermediate-types	01:08	01:37			
	Morning-types	23:58	01:17			
FWT	Evening-types	10:10	01:41	**18.25**	**0.0001**	**0.05**
	Intermediate-types	09:13	01:38			
	Morning-types	08:14	01:27			
FTIB	Evening-types	07:57	01:35	2.53	0.08	0.01
	Intermediate-types	08:05	01:26			
	Morning-types	08:16	01:16			
FMPoS	Evening-types	06:11	01:25	**34.45**	**0.0001**	**0.09**
	Intermediate-types	05:10	01:28			
	Morning-types	04:06	01:13			
SJL	Evening-types	01:47	01:50	0.64	0.53	0.002
	Intermediate-types	01:35	01:08			
	Morning-types	01:17	01:03			
PE	Evening-types	0.23	0.81	2.34	0.10	0.01
	Intermediate-types	0.42	0.64			
	Morning-types	0.43	0.72			
PT	Evening-types	0.80	0.69	0.48	0.62	0.001
	Intermediate-types	0.79	0.70			
	Morning-types	0.86	0.70			
TP	Evening-types	3.62	0.63	1.09	0.34	0.003
	Intermediate-types	3.48	0.72			
	Morning-types	3.46	0.77			
TE/B	Evening-types	2.64	0.82	**6.40**	**0.002**	**0.02**
	Intermediate-types	2.45	0.81			
	Morning-types	2.20	0.85			
Total PRMQ score	Evening-types	46.45	11.75	**14.80**	**0.0001**	**0.04**
	Intermediate-types	50.39	10.43			
	Morning-types	53.56	11.10			
Pro score	Evening-types	45.85	12.72	**12.50**	**0.0001**	**0.04**
	Intermediate-types	49.69	10.77			
	Morning-types	52.09	11.02			
Retro score	Evening-types	46.09	9.94	**13.94**	**0.0001**	**0.04**
	Intermediate-types	49.50	9.77			
	Morning-types	52.97	10.49			

The F, p, and partial eta-squared ($\eta_{\text{p}}^{2}$) for circadian typology factor are also shown. The significant results are shown in bold.

Sleep factor, Mini-Sleep Questionnaire Sleep factor; Wake factor, Mini-Sleep Questionnaire Wake factor; WBT, Working Bed Time; WWT, Working Wake Time; WTIB, Working Time In Bed; WMPoS, Working Mid-Point of Sleep; FBT, Free Bed Time; FWT, Free Wake Time; FTIB, Free Time In Bed; FMPoS, Free Mid-Point of Sleep; SJL, Social JetLag; PE, Present Experience; PT, Past Time; TP, Time Pressure; TE/B, Time Expansion/Boredom; PRMQ, Prospective-Retrospective Memory Questionnaire for total score; PRO, Prospective score; RETRO, Retrospective score.

Table 3 presents the correlation matrix among all variables (see Supplementary materials for assessing the same correlation matrix separately for each chronotype). Total PRMQ scores, along with the Prospective (Pro) and Retrospective (Retro) subscores, showed significant associations with multiple variables. Specifically, better memory performance was associated with a morningness chronotype, fewer sleep and wake problems, earlier bedtimes and wake times on workdays or university days, and earlier midpoints of sleep (MPoS). In addition, lower levels of perceived time pressure and less frequent feelings of time expansion or boredom were related to better memory outcomes. More selectively, earlier bedtimes on free days and earlier MPoS on free days were significantly associated with higher Retro scores, as well as total PRMQ scores, further indicating that more regular and earlier sleep-wake patterns may support better memory functioning.

**Table 3:** The *r* values of correlation coefficients are reported above the major diagonal of the correlation matrix.

	**1**	**2**	**3**	**4**	**5**	**6**	**7**	**8**	**9**	**10**	**11**	**12**	**13**	**14**	**15**	**16**	**17**	**18**	**19**
1-rMEQ	**1**	**−0.20** ^*^	**−0.34** ^*^	**−0.39** ^*^	**−0.27** ^*^	+0.08	**−0.38** ^*^	**−0.35** ^*^	**−0.30** ^*^	+0.06	**−0.38** ^*^	−0.05	+0.07	+0.02	−0.09	**−0.20** ^*^	**+0.21** ^*^	**+0.20** ^*^	**+0.19** ^*^
2-MSQ Sleep	–	**1**	**+0.58** ^*^	**+0.19** ^*^	+0.08	−0.09	**+0.15** ^*^	+0.09	−0.04	**−0.13°**	+0.03	**−0.11°**	−0.01	−0.02	**+0.14** ^*^	**+0.25** ^*^	**−0.26** ^*^	**−0.25** ^*^	**−0.24** ^*^
3-MSQ Wake	–	–	**1**	**+0.20** ^*^	+0.09	−0.08	**+0.17** ^*^	**+0.11°**	+0.001	**−0.11°**	+0.07	−0.09	−0.03	−0.002	**+0.26** ^*^	**+0.29** ^*^	**−0.39** ^*^	**−0.38** ^*^	**−0.34** ^*^
4-WBT	–	–	–	**1**	**+0.43** ^*^	**−0.46** ^*^	**+0.83** ^*^	**+0.54** ^*^	**+0.27** ^*^	**−0.27** ^*^	**+0.47** ^*^	**−0.28** ^*^	−0.03	−0.01	+0.06	**+0.14** ^*^	**−0.14** ^*^	**−0.14** ^*^	**−0.13** ^*^
5-WWT	–	–	–	–	**1**	**+0.60** ^*^	**+0.86** ^*^	**+0.29** ^*^	**+0.46** ^*^	**+0.16** ^*^	**+0.44** ^*^	**−0.36** ^*^	−0.02	−0.001	−0.06	**+0.25** ^*^	**−0.14** ^*^	**−0.11°**	**−0.14** ^*^
6-WTIB	–	–	–	–	–	**1**	**+0.12** ^*^	**−0.19** ^*^	**+0.21** ^*^	**+0.39** ^*^	+0.01	**−0.10°**	+0.003	+0.01	**−0.11°**	**+0.12°**	−0.01	+0.01	−0.03
7-WMPoS	–	–	–	–	–	–	**1**	**+0.48** ^*^	**+0.44** ^*^	−0.05	**+0.53** ^*^	**−0.38** ^*^	−0.03	−0.01	−0.01	**+0.23** ^*^	**−0.16** ^*^	**−0.15** ^*^	**−0.16** ^*^
8-FBT	–	–	–	–	–	–	–	**1**	**+0.49** ^*^	**−0.51** ^*^	**+0.87** ^*^	**+0.48** ^*^	+0.02	+0.02	+0.05	+0.06	**−0.11°**	−0.08	**−0.14** ^*^
9-FWT	–	–	–	–	–	–	–	–	**1**	**+0.50** ^*^	**+0.86** ^*^	**+0.52** ^*^	+0.004	+0.08	−0.06	**+0.12°**	−0.06	−0.04	−0.09
10-FTIB	–	–	–	–	–	–	–	–	–	**1**	−0.01	+0.04	−0.01	+0.06	**−0.11°**	+0.06	+0.05	+0.05	+0.05
11-FMPoS	–	–	–	–	–	–	–	–	–	–	**1**	**+0.58** ^*^	+0.01	+0.06	−0.003	**+0.11°**	**−0.10°**	−0.07	**−0.13°**
12-SJL	–	–	–	–	–	–	–	–	–	–	–	**1**	+0.04	+0.07	+0.005	**−0.11°**	+0.04	+0.07	+0.01
13-PE	–	–	–	–	–	–	–	–	–	–	–	–	**1**	**+0.31** ^*^	**+0.15** ^*^	**−0.22** ^*^	−0.02	+0.002	−0.04
14-PT	–	–	–	–	–	–	–	–	–	–	–	–	–	**1**	**−0.17** ^*^	**−0.15** ^*^	+0.05	+0.06	+0.04
15-TP	–	–	–	–	–	–	–	–	–	–	–	–	–	–	**1**	**−0.15** ^*^	**−0.23** ^*^	**−0.24** ^*^	**−0.18** ^*^
16-TE/B	–	–	–	–	–	–	–	–	–	–	–	–	–	–	–	**1**	**−0.26** ^*^	**−23** ^*^	**−0.26** ^*^
17-PRMQ	–	–	–	–	–	–	–	–	–	–	–	–	–	–	–	–	**1**	**+0.94** ^*^	**+0.94** ^*^
18-PRO	–	–	–	–	–	–	–	–	–	–	–	–	–	–	–	–	–	**1**	**+0.77** ^*^
19-RETRO	–	–	–	–	–	–	–	–	–	–	–	–	–	–	–	–	–	–	**1**

In bold are the significant correlations.

rMEQ, reduced Morningness-Eveningness Questionnaire; MSQ Sleep, Mini-Sleep Questionnaire for Sleep factor; MSQ Wake, Mini-Sleep Questionnaire for Wake factor; WBT, Working Bed Time; WWT, Working Wake Time; WTIB, Working Time In Bed; WMPoS, Working Mid-Point of Sleep; FBT, Free Bed Time; FWT, Free Wake Time; FTIB, Free Time In Bed; FMPoS, Free Mid-Point of Sleep; SJL, Social JetLag; PE, Present Experience; PT, Past Time; TP, Time Pressure; TE/B, Time Expansion/Boredom; PRMQ, Prospective-Retrospective Memory Questionnaire for total score; PRO, Prospective score; RETRO, Retrospective score. In addition, °p **≤** 0.01 and ^*^p ≤ 0.001.

To identify which variables most strongly predicted memory performance, three stepwise linear regressions were conducted. The first regression model focused on the PRMQ total score and yielded a significant model with a *R*^2^_*adjusted*_ = 0.20, *F*_(3, 662)_ = 56.50, *p* = 0.0001. The most significant predictors were the MSQ Wake Factor (*b* = −0.64, *t* = −7.29, *p* = 0.0001, 95% CI [−0.81, −0.47]), Time Expansion/Boredom (TE/B; *b* = −2.59, *t* = −5.27, *p* = 0.0001, 95% CI [−3.56, −1.63]), and Time Pressure (TP; *b* = −2.77, *t* = −4.94, *p* = 0.0001, 95% CI [−3.88, −1.67]). The second regression, using the Prospective memory subscale (Pro) as the outcome, also revealed a significant model with a *R*^2^_*adjusted*_ = 0.20, *F*_(4, 661)_ = 42.09, *p* = 0.0001. Four variables emerged as significant predictors: the MSQ Wake Factor (*b* = −0.66, *t* = −7.32, *p* = 0.0001, 95% CI [−0.84, −0.48]), TP (*b* = −3.03, *t* = −5.25, *p* = 0.0001, 95% CI [−4.16, −1.90]), TE/B (*b* = −2.05, *t* = −4.06, *p* = 0.0001, 95% CI [−3.04, −1.06]), and Social Jetlag (SJL; *b* = +0.92, *t* = 2.89, *p* = 0.004, 95% CI [+0.30, +1.55]). The third regression, predicting Retrospective memory (Retro), was also significant, with a *R*^2^_*adjusted*_ = 0.17, *F*_(4, 661)_ = 34.70, *p* = 0.0001. Predictors in this model included the MSQ Wake Factor (*b* = −0.51, *t* = −6.16, *p* = 0.0001, 95% CI [−0.68, −0.35]), TE/B (*b* = −2.79, *t* = −5.87, *p* = 0.0001, 95% CI [−3.73, −1.86]), TP (*b* = −1.99, *t* = −3.71, *p* = 0.0001, 95% CI [−3.04, −0.94]), and Present Experience (PE; *b* = −1.07, *t* = −1.97, *p* = 0.049, 95% CI [−2.13, −0.005]), although the latter effect did not reach statistical significance.

Based on these types of statistical analyses, three mediation models were tested to examine the indirect effects of chronotype on memory via sleep-wake functioning and time perception. In each model, rMEQ scores served as the predictor variable, the MSQ Wake Factor as the first-level mediator, and TP and TE/B as second-level mediators. Memory scores (PRMQ, Pro, or Retro) were treated as dependent variables, while age and gender were included as covariates. The model predicting PRMQ was statistically significant, *R*^2^ = 0.23, *F*_(6, 659)_ = 32.83, *p* = 0.00001. No direct effect of rMEQ on PRMQ was found (β = +0.21, *p* = 0.07, 95% CI [−0.02, +0.44]), but several significant indirect effects were observed. Specifically, higher rMEQ scores predicted lower Wake scores, which in turn predicted better memory (β = +0.27, 95% CI [+0.17, +0.39]). A similar indirect path emerged through TE/B (β = +0.08, 95% CI [+0.02, +0.14]). Additional significant indirect effects included: rMEQ → Wake → TP → PRMQ (β = +0.06, 95% CI [+0.03, +0.08]), rMEQ → Wake → TE/B → PRMQ (β = +0.07, 95% CI [+0.04, +0.10]), and a smaller but significant three-step pathway from rMEQ through Wake, TP, and TE/B to PRMQ (β = −0.01, 95% CI [−0.02, −0.001]). These mediation patterns were also observed in the models predicting the Pro and Retro subscores. The model for Pro was significant, *R*^2^ = 0.24, *F*_(6, 659)_ = 34.35, *p* = 0.00001, and the model for Retro was also significant, *R*^2^ = 0.18, *F*_(6, 659)_ = 23.57, *p* = 0.00001. The findings suggest that the influence of circadian preference on memory is at least partially mediated by daytime alertness and subjective experiences of time, such as perceived time pressure and boredom.

## 4 Discussion

The present study aimed to investigate, for the first time, how biological time (measured via circadian typology) and psychological time (indexed by time awareness and subjective time perception) contribute to memory failures, accounting for sleep-wake timing and quality. Using both categorical and continuous statistical approaches, with conservative thresholds for significance, we identified specific predictors of PRMQ scores. Specifically, the present study mainly showed that morningness-eveningness preference, wake problem (e.g., daytime sleepiness), and time pressure or time expansion/boredom were constantly related to retrospective and prospective memory functioning. This explorative study seems to indicate a possible intersection between circadian biology, sleep-wake quality and time perception for explaining memory functioning.

Circadian typology was significantly associated with memory functioning (Fabbri et al., 2013; Palmero et al., 2024; Petros et al., 1990; Rothen and Meier, 2017). Morning-types reported better memory performance, whereas evening-types reported more frequent memory slips, confirming the role of circadian rhythms in long-term memory (Kelley et al., 2018; Smarr et al., 2014; Taillard et al., 2021). Taking into account that PRMQ is mainly focused on episodic memory, our results could indicate that morningness-eveningness preference is a factor to be considered on the base of the long-term memory system, given that the previous studies basically reported similar memory performance in extreme chronotypes for semantic memory (Fabbri et al., 2013; Palmero et al., 2024; Petros et al., 1990; Rothen and Meier, 2017). Probably, the different memory performance between morning- and evening-types reported in the present study could be related to different circadian misalignment between chronotypes (Cho, 2001; Eckel-Mahan et al., 2008; Smies et al., 2022). Evening-types showed delayed BT, WT, and MPoS across both workdays and free days (Roenneberg et al., 2007; Taillard et al., 2021; Wittmann et al., 2006), indicating variability in the phase angle of entrainment (Duffy et al., 1999; Duffy and Wright, 2005). This delay could reflect a misalignment where evening-types wake at a later local time but at an earlier circadian phase, resulting in increased effort during wakefulness (Mongrain et al., 2006; Taillard et al., 2021). Correlational analyses (Table 3) showed that later sleep-wake times (WBT, WWT, WMPoS, FBT, FWT, FMPoS) were systematically associated with poorer memory functioning. Additionally, evening-types reported poorer sleep quality and greater daytime sleepiness (Table 2), which may impair encoding and retrieval processes (Barner et al., 2017; Chambers, 2017; Diekelmann and Born, 2010; Ellenbogen et al., 2009; Esposito et al., 2015; Fabbri et al., 2014, 2015; Feld et al., 2016; Fenn et al., 2009; Kim et al., 2022; Klinzing et al., 2019; Krause et al., 2017; Leong et al., 2019; Occhionero et al., 2020; Rasch and Born, 2013; Scullin and McDaniel, 2010; van der Helm et al., 2011). When correlational analyses were conducted separately for each chronotype, distinct patterns emerged (see Supplementary materials). Among evening types, memory functioning was positively associated with wake time on free days, suggesting that better retrospective and prospective memory performance was linked to later wake timing. This relationship may reflect an alignment between memory efficiency and the chronotype's biological rhythms, potentially mediated by reduced daytime sleepiness (Roenneberg et al., 2007; Taillard et al., 2021). In intermediate types, PRMQ scores were negatively associated both with sleep–wake quality and with altered subjective experience of time. Specifically, memory errors increased with poorer sleep–wake regulation and with stronger feelings of time pressure or time distortion (e.g., expansion or boredom). A comparable pattern was observed in morning types, with the exception that no significant associations emerged between PRMQ scores and either the MSQ sleep factor or the TE/B dimension. The role of sleep–wake quality in shaping memory functioning is further discussed below. With regard to the perception of time, it is noteworthy that in morning types PRMQ scores correlated with time pressure. This finding aligns with evidence that morningness is associated with stronger future orientation (Beracci et al., 2022). It is possible that a heightened sense of time pressure, while fostering future-oriented tendencies, may simultaneously reduce the processing of non-temporal information, thereby increasing susceptibility to memory errors.

Consistently, both MSQ sleep and wake scores negatively correlated with PRMQ scores, indicating that poorer sleep and increased daytime sleepiness predicted higher memory errors. Although we recognize the correlational design of our study, the fact that eveningness was associated with sleep-wake problems could suggest that the memory deficits in evening-types may also relate to their homeostatic and circadian regulation of alertness (Adan et al., 2012; Chauhan et al., 2025; May et al., 2023; Wiłkość-Dȩbczyǹska and Liberacka-Dwojak, 2023). It is possible to advance, at speculative level, that the SCN, via the locus coeruleus (LC), modulates cortical activation, particularly in prefrontal regions involved in memory (Addis et al., 2009; Aston-Jones, 2005; Leon and Shadlen, 2003; Meck et al., 2008; Rolls, 2022). A suboptimal circadian profile may impair alertness, reducing memory encoding accuracy (Lozito and Mulligan, 2006). Although speculative, it can be recognized the contribution of the genetic variability in clock genes (e.g., Kalmbach et al., 2017), as longer intrinsic periods are associated with delayed circadian phases (Taillard et al., 2021). Future studies should examine these mechanisms using neuroscientific methods (Kelley et al., 2018).

Regression analyses showed that MSQ wake scores, time expansion/boredom (TE/B), and time pressure (TP) were consistent predictors of PRMQ scores. Based on the wide literature showing a sleep effect on memory (Barner et al., 2017; Chambers, 2017; Diekelmann and Born, 2010; Diekelmann et al., 2013; Esposito et al., 2015; Fabbri et al., 2014, 2015; Girardeau and Lopes-Dos-Santos, 2021; Klinzing et al., 2019; Leong et al., 2019; Occhionero et al., 2020; Rasch and Born, 2013; Scullin and McDaniel, 2010; Tonetti et al., 2020; de Vivo et al., 2017), the first predictor was unexpected or, at least, an influence of MSQ sleep problem should be expected. From one hand, higher scores at MSQ wake factor reflect daytime sleepiness which, in turn, could reduce attentional resources and/or alertness levels, impairing memory formation, consistent with findings from sleep deprivation literature (Casini et al., 2013; Sen et al., 2023; Soshi et al., 2010). This aligns with the wake-state instability hypothesis (Doran et al., 2001), suggesting fluctuations in neurocognitive performance due to elevated homeostatic pressure and reduced alertness. According to this hypothesis, when alertness is low, long-term memory encoding is impaired (Fields, 2005). On the other hand, we acknowledge that we adopted self-report measure of daily memory errors administered during the day, while the majority of the studies has adopted experimental procedure, during one or more sleep nights, in controlled laboratory with objective measures of both sleep-wake cycle and memory tasks. These differences could explain our data and future studies should use experimental paradigms in laboratory setting with objective measures in order to extend our results in a more controlled setting. The other systematic predictors of memory errors were TE/B and TP, as indices of psychological time, suggesting that distorted perception of time's passage (Beracci et al., 2022; Droit-Volet and Martinelli, 2023; Martinelli and Droit-Volet, 2022; Wearden, 2015; Wittmann et al., 2015) impacts memory accuracy. A misjudged passage of time may divert cognitive resources from non-temporal information, impairing encoding and information processing. This aligns with findings on the interplay between arousal, attention, alertness and time perception (Block et al., 2010; Block and Gruber, 2014; Glicksohn, 2001; Matthews and Meck, 2016). For instance, it has been reported that high arousal accelerates subjective time, while low arousal slows it (Droit-Volet and Wearden, 2016). This change (both accelerating and slowing down) in the feeling of subjective passage of time could potentially affect long-term memory encoding (Fields, 2005; Kelley and Whatson, 2013). For example, boredom, a state of suboptimal arousal, disrupts attentional focus (Eastwood et al., 2012; Carriere et al., 2008; Gerritsen et al., 2014; Zakay, 2014). It has been reported that boredom seems to activate medial prefrontal regions and the DMN while it deactivates memory-related areas such as the hippocampus (Raffaelli et al., 2018). This may explain the link between TE/B and memory errors. TP may exert similar effects via attentional shifts toward temporal cues and the recruitment of overlapping neural systems involved in both time perception and memory (Addis et al., 2009; Coslett et al., 2009; Leon and Shadlen, 2003; Meck et al., 2008; Miloyan et al., 2019; Muller and Nobre, 2014; Nyberg et al., 2010; Rolls, 2022). In other words, the feeling that the “*time runs away*” could capture the attentional resources (and the activation of specific areas for the time perception) limiting an efficiency encoding of information with the possibility of an increase of memory errors. Given that our study, for the first time, reports the involvement of psychological time for memory performance, future studies are needed.

**Figure 1:**
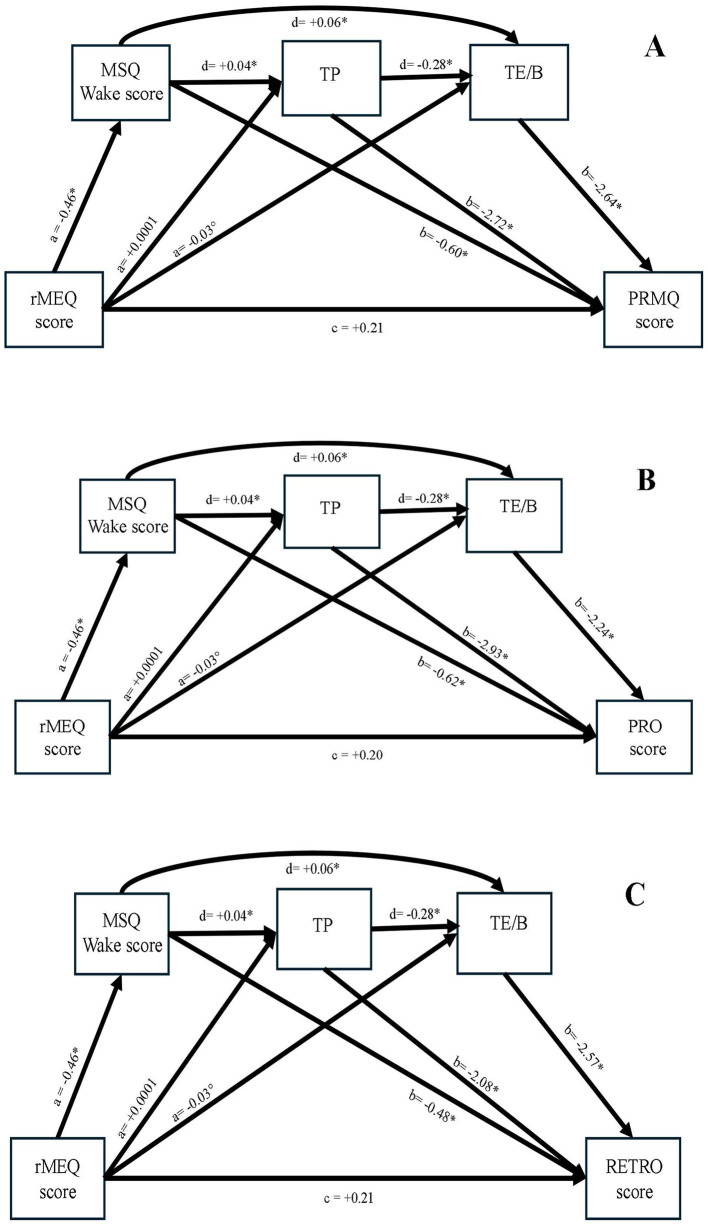
The model illustrates the role of circadian typology in predicting the PRMQ score **(A)**, Pro score **(B)**, and Retro score **(C)** both directly and indirectly via MSQ Wake score, time pressure (TP), and time expansion/boredom (TE/B). *p < 0.00001 while °*p* < 0.01.

Mediation analyses further elucidated possible mechanisms linking biological and psychological time to memory functioning (Figure 1). First, eveningness predicted increased daytime sleepiness, which in turn predicted poorer memory performance, paralleling effect observed in experimental (Ellenbogen et al., 2009; Feld et al., 2016; Fenn et al., 2009; Kim et al., 2022; Krause et al., 2017; van der Helm et al., 2011). Second, eveningness was associated with TE/B, which predicted memory slips. Evening-types may experience a slower POT due to reduced attentional engagement and low alertness (Beracci et al., 2022). Boredom or time expansion can decrease present-moment awareness (Fabbri, 2023; Raffaelli et al., 2018), lowering non-temporal information processing and leading to memory errors. A third indirect pathway revealed that eveningness was linked to both sleep-wake problems and altered POT (TE/B and TP), which predicted higher memory errors. This suggests that circadian misalignment leads to attentional impairments and altered time perception, ultimately reducing memory accuracy. These findings align with previous work showing that sleepiness distorts time perception (Casini et al., 2013; Zakay, 2014) and affects retrospective and prospective memory (Beracci et al., 2022; Doran et al., 2001; Esposito et al., 2007). In sum, evening-types appear more vulnerable to memory failures due to the combined effects of circadian misalignment, daytime sleepiness, and altered time perception. Future experimental studies are needed to replicate and extend this model in laboratory settings.

This cross-sectional design does not allow causal inference. The proposed explanations or possible mechanisms advanced are based on the integrations of what has been reported in the literature with our cross-sectional data. Thus, longitudinal and experimental studies are needed. Memory was self-reported; future work should employ objective tasks. Indeed, subjective measures of memory functioning could determine underestimation or overestimation of memory performance, for example, due to a bias in retrieval of cases equal or similar to those reported in the PRMQ. Although we used reliable and valid tool for assessing memory errors in daily live, objective memory task could measure the individual memory functioning for different long-term memory system More precise measures of chronotype (e.g., melatonin profiling or genotyping) and physiological indicators of alertness (e.g., EEG) would enhance validity. At the same time, the use of forced desynchrony paradigm with an artificial sleep-wake cycle or the constant routine paradigm (Schmidt et al., 2007) could analyze the impact of homeostatic and circadian processes in time perception and in memory functioning. Alternative tasks to assess POT (e.g., Beracci et al., 2022) should also be considered, such as time estimation task, which allows to measure the “correct” functioning of pacemaker. The sample was unbalanced in terms of gender and age and recruited via convenience sampling, limiting generalizability. Lastly, the time of day at questionnaire completion was not recorded, preventing analysis of time-of-day or synchrony effects (Chauhan et al., 2025; May et al., 2023). Future studies should incorporate paradigms such as constant routine or forced desynchrony (Schmidt et al., 2007; Taillard et al., 2021) to assess the impact of homeostatic and circadian processes.

This large-sample cross-sectional study is the first to show that circadian rhythms, sleep-wake quality, and psychological time perception independently and jointly predict memory functioning. Evening-types, more prone to circadian misalignment, experienced greater daytime sleepiness, altered alertness levels (and arousal and attention), distorted time perception (TP and TE/B), and more frequent memory errors. Given the prevalence of eveningness in adolescence (Taillard et al., 2021) and its relevance to educational contexts (Kelley et al., 2018), further research should explore the biological and psychological mechanisms through which internal and subjective time influence memory.

## Data Availability

The raw data supporting the conclusions of this article will be made available by the authors, without undue reservation.
