# Supplementary material for: How chronotype, sleep-wake cycle, subjective time experience influence retrospective, and prospective memory functioning
Source: Front Cognit. 2025 Oct 17;4:1683207. doi: 10.3389/fcogn.2025.1683207 (PMC13281119; doi:10.3389/fcogn.2025.1683207)
Supplement: Supplementary file 1 [file Data_Sheet_1.docx]

**Supplementary Material**

Three additional partial correlation analyses, controlling for age and gender, were conducted to examine the associations among all variables, separately for each chronotype. This approach allowed us to investigate how the relationships between variables varied according to chronotype. The corresponding correlation coefficients are reported in Tables S1, S2, and S3.

For evening-types (Table S1), we largely confirmed and extended the associations between sleep–wake habits, social jetlag (SJL), and sleep–wake problems, underscoring the importance of both sleep–wake regularity and sleep–wake timing. We observed positive associations between TE/B and the Wake factor of the MSQ, as well as with wake-up time on work/university days. This suggests that individuals with wake-related difficulties or a delayed waking time on weekdays tend to experience greater time expansion or boredom, likely due to excessive daytime sleepiness. Furthermore, we found positive associations between all PRMQ scales and wake-up time on free days, along with a specific positive association between the prospective scale and SJL. These findings highlight the importance of aligning daily routines with one’s biological rhythm to support cognitive performance.

For intermediate-types (Table S2), we replicated the associations between sleep–wake habits, SJL, and sleep problems (with the sole exception of wake problems and TIB on free days). In addition, we identified positive associations between sleep–wake problems and subjective perception of the passage of time (both TP and TE/B), as well as negative associations between sleep–wake problems and all PRMQ scales. Thus, for intermediate-types, individuals with sleep and/or wake difficulties reported both time pressure and time expansion. Moreover, disturbances in the sleep–wake cycle were linked to reduced retrospective and prospective memory performance. Interestingly, in this subsample, we also found negative associations between TP or TE/B and all PRMQ scales, suggesting a connection between subjective perception of time passage and memory functioning.

For morning-types (Table S3), we essentially replicated the pattern of associations between sleep–wake variables observed in the other two chronotypes. Additionally, we found positive associations between the MSQ Wake factor and both TP and TE/B, as well as negative correlations between wake problems and all PRMQ scales, reinforcing the idea that daytime sleepiness is related to altered time perception and increased memory errors. In this chronotype, only TP was negatively associated with PRMQ, indicating that a faster subjective passage of time was linked to poorer retrospective and prospective memory performance.

**Table S1.** The r values of correlation coefficients when only evening-types are selected, are reported above the major diagonal of the correlation matrix. In bold are the significant correlations.

|  | 1 | 2 | 3 | 4 | 5 | 6 | 7 | 8 | 9 | 10 | 11 | 12 | 13 | 14 | 15 | 16 | 17 | 18 | 19 |
| --- | --- | --- | --- | --- | --- | --- | --- | --- | --- | --- | --- | --- | --- | --- | --- | --- | --- | --- | --- |
| 1-rMEQ | **1** | +.21 | -.02 | +.09 | -.03 | -.12 | +.03 | -.14 | **-.41*** | -.24 | **-.32°** | -.25 | +.12 | +.08 | -.07 | -.15 | -.04 | -.07 | -.01 |
| 2-MSQ Sleep | - | **1** | **+.49*** | +.10 | +.04 | -.05 | +.07 | -.14 | **-.35*** | -.18 | -.28 | -.26 | +.11 | +.28 | +.07 | +.21 | -.25 | -.27 | -.22 |
| 3-MSQ Wake | - | - | **1** | +.27 | +.21 | -.01 | +.27 | **-.35*** | -.16 | +.17 | -.29 | **-.41*** | -.13 | +.22 | +.12 | **+.33*** | -.26 | -.29 | -.19 |
| 4-WBT | - | - | - | **1** | **+.52*** | **-.32°** | **+.84*** | -.001 | -.003 | -.002 | -.003 | **-.63*** | -.07 | +.06 | -.13 | +.13 | -.24 | -.31 | -.12 |
| 5-WWT | - | - | - | - | **1** | **+.64*** | **+.90*** | +.002 | +.18 | +.16 | +.10 | **-.60*** | +.02 | +.002 | -.20 | **+.38*** | -.01 | -.04 | +.03 |
| 6-WTIB | - | - | - | - | - | **1** | +.24 | +.004 | +.20 | +.18 | +.12 | -.10 | +.08 | -.05 | -.10 | +.30 | +.20 | +.23 | +.14 |
| 7-WMPoS | - | - | - | - | - | - | **1** | +.001 | +.11 | +.10 | +.06 | **-.70*** | -.03 | +.03 | -.19 | +.31 | -.13 | -.19 | -.04 |
| 8-FBT | - | - | - | - | - | - | - | **1** | **+.44*** | **-.56*** | **+.86*** | **+.61*** | -.03 | -.25 | -.07 | -.08 | +.09 | +.13 | +.05 |
| 9-FWT | - | - | - | - | - | - | - | - | **1** | **+.49*** | **+.84*** | **+.52*** | -.07 | -.13 | -.04 | -.04 | **+.41*** | **+.41*** | **+.38*** |
| 10-FTIB | - | - | - | - | - | - | - | - | - | **1** | -.06 | -.12 | -.04 | +.13 | +.03 | +.05 | +.29 | +.25 | +.30 |
| 11-FMPoS | - | - | - | - | - | - | - | - | - | - | **1** | **+.67*** | -.06 | -.23 | -.06 | -.07 | +.29 | +.31 | +.25 |
| 12-SJL | - | - | - | - | - | - | - | - | - | - | - | **1** | -.02 | -.18 | +.10 | -.28 | +.30 | **+.36*** | +.21 |
| 13-PE | - | - | - | - | - | - | - | - | - | - | - | - | **1** | +.22 | +.22 | -.30 | -.003 | +.05 | -.08 |
| 14-PT | - | - | - | - | - | - | - | - | - | - | - | - | - | **1** | +.26 | +.04 | -.03 | -.03 | -.02 |
| 15-TP | - | - | - | - | - | - | - | - | - | - | - | - | - | - | **1** | -.21 | -.17 | -.16 | -.16 |
| 16-TE/B | - | - | - | - | - | - | - | - | - | - | - | - | - | - | - | **1** | -.30 | -.27 | -.30 |
| 17-PRMQ | - | - | - | - | - | - | - | - | - | - | - | - | - | - | - | - | **1** | **+.96*** | **+.95*** |
| 18-PRO | - | - | - | - | - | - | - | - | - | - | - | - | - | - | - | - | - | **1** | **+.83*** |
| 19-RETRO | - | - | - | - | - | - | - | - | - | - | - | - | - | - | - | - | - | - | **1** |

Note. rMEQ for reduced Morningness-Eveningness Questionnaire, MSQ Sleep for Mini-Sleep Questionnaire for Sleep factor, MSQ Wake for Mini-Sleep Questionnaire for Wake factor, WBT for Working Bed Time, WWT for Working Wake Time, WTIB for Working Time In Bed, WMPoS for Working Mid-Point of Sleep, FBT for Free Bed Time, FWT for Free Wake Time, FTIB for Free Time In Bed, FMPoS for Free Mid-Point of Sleep, SJL for Social JetLag, PE for Present Experience, PT for Past Time, TP for Time Pressure, TE/B for Time Expansion/Boredom, PRMQ for Prospective-Retrospective Memory Questionnaire for total score, PRO for Prospective Score and RETRO for Retrospective Score. In addition, °*p* = .01 and **p*≤ .001

**Table S2.** The r values of correlation coefficients when only intermediate-types are selected, are reported above the major diagonal of the correlation matrix. In bold are the significant correlations.

|  | 1 | 2 | 3 | 4 | 5 | 6 | 7 | 8 | 9 | 10 | 11 | 12 | 13 | 14 | 15 | 16 | 17 | 18 | 19 |
| --- | --- | --- | --- | --- | --- | --- | --- | --- | --- | --- | --- | --- | --- | --- | --- | --- | --- | --- | --- |
| 1-rMEQ | **1** | -.10 | **-.22*** | **-.24*** | **-.19*** | +.02 | **-.25*** | **-.21*** | **-.22*** | -.01 | **-.26*** | -.04 | +.06 | +.01 | -.08 | **-.21*** | +.10 | +.11 | +.08 |
| 2-MSQ Sleep | - | **1** | **+.56*** | **+.16*** | +.04 | -.10 | +.12 | +.07 | -.08 | **-.14*** | -.004 | -.12 | -.09 | -.08 | **+.14*** | **+.27*** | **-.25*** | **-.23*** | **-.23*** |
| 3-MSQ Wake | - | - | **1** | +.10 | -.002 | -.08 | +.05 | +.09 | -.05 | **-.14*** | +.02 | -.03 | -.04 | -.04 | **+.29*** | **+.25*** | **-.36*** | **-.36*** | **-.32*** |
| 4-WBT | - | - | - | **1** | **+.40*** | **-.46*** | **+.81*** | **+.55*** | **+.26*** | **-.28*** | **+.47*** | **-.26*** | +.01 | -.004 | +.05 | +.12 | -.09 | -.08 | -.09 |
| 5-WWT | - | - | - | - | **1** | **+.63*** | **+.86*** | **+.31*** | **+.48*** | **+.16*** | **+.47*** | **-.32*** | -.03 | +.004 | -.05 | **+.22*** | -.11 | -.08 | -.12 |
| 6-WTIB | - | - | - | - | - | **1** | **+.15*** | **-.16*** | **+.25*** | **+.39*** | +.05 | -.09 | -.04 | +.007 | -.09 | +.12 | -.03 | -.01 | -.03 |
| 7-WMPoS | - | - | - | - | - | - | **1** | **+.50*** | **+.45*** | **-.05** | **+.56*** | **-.35*** | -.01 | +.0001 | -.007 | **+.21*** | -.12 | -.10 | **-.13*** |
| 8-FBT | - | - | - | - | - | - | - | **1** | **+.45*** | **-.53*** | **+.85*** | **+.47*** | +.06 | +.07 | +.04 | +.04 | -.09 | -.07 | -.10 |
| 9-FWT | - | - | - | - | - | - | - | - | **1** | **+.52*** | **+.85*** | **+.52*** | +.04 | +.12 | -.08 | +.09 | -.05 | -.04 | -.07 |
| 10-FTIB | - | - | - | - | - | - | - | - | - | **1** | -.007 | +.05 | -.03 | +.05 | -.12 | +.05 | +.04 | +.03 | +.04 |
| 11-FMPoS | - | - | - | - | - | - | - | - | - | - | **1** | **+.58*** | +.06 | +.11 | -.02 | +.08 | -.08 | -.06 | -.09 |
| 12-SJL | - | - | - | - | - | - | - | - | - | - | - | **1** | +.08 | **+.12°** | -.02 | -.12 | +.03 | +.03 | +.01 |
| 13-PE | - | - | - | - | - | - | - | - | - | - | - | - | **1** | **+.34*** | +.11 | **-.20*** | +.03 | +.03 | +.02 |
| 14-PT | - | - | - | - | - | - | - | - | - | - | - | - | - | **1** | +.12 | **-.20*** | +.10 | +.10 | +.09 |
| 15-TP | - | - | - | - | - | - | - | - | - | - | - | - | - | - | **1** | -.09 | **-.22*** | **-.24*** | **-.16*** |
| 16-TE/B | - | - | - | - | - | - | - | - | - | - | - | - | - | - | - | **1** | **-.26*** | **-.22*** | **-.27*** |
| 17-PRMQ | - | - | - | - | - | - | - | - | - | - | - | - | - | - | - | - | **1** | **+.94*** | **+.93*** |
| 18-PRO | - | - | - | - | - | - | - | - | - | - | - | - | - | - | - | - | - | **1** | **+.74*** |
| 19-RETRO | - | - | - | - | - | - | - | - | - | - | - | - | - | - | - | - | - | - | **1** |

Note. rMEQ for reduced Morningness-Eveningness Questionnaire, MSQ Sleep for Mini-Sleep Questionnaire for Sleep factor, MSQ Wake for Mini-Sleep Questionnaire for Wake factor, WBT for Working Bed Time, WWT for Working Wake Time, WTIB for Working Time In Bed, WMPoS for Working Mid-Point of Sleep, FBT for Free Bed Time, FWT for Free Wake Time, FTIB for Free Time In Bed, FMPoS for Free Mid-Point of Sleep, SJL for Social JetLag, PE for Present Experience, PT for Past Time, TP for Time Pressure, TE/B for Time Expansion/Boredom, PRMQ for Prospective-Retrospective Memory Questionnaire for total score, PRO for Prospective Score and RETRO for Retrospective Score. In addition, °*p* = .01 and **p*≤ .001

**Table S3.** The r values of correlation coefficients when only morning-types are selected, are reported above the major diagonal of the correlation matrix. In bold are the significant correlations.

|  | 1 | 2 | 3 | 4 | 5 | 6 | 7 | 8 | 9 | 10 | 11 | 12 | 13 | 14 | 15 | 16 | 17 | 18 | 19 |
| --- | --- | --- | --- | --- | --- | --- | --- | --- | --- | --- | --- | --- | --- | --- | --- | --- | --- | --- | --- |
| 1-rMEQ | **1** | -.11 | -.05 | -.13 | -.06 | +.05 | -.11 | -.11 | -.18 | -.09 | -.17 | -.09 | -.07 | -.02 | -.13 | +.008 | -.02 | +.03 | -.06 |
| 2-MSQ Sleep | - | **1** | **+.60*** | +.07 | +.08 | +.02 | +.09 | +.02 | +.04 | +.03 | +.04 | -.04 | +.20 | +.06 | +.12 | +.16 | -.18 | -.16 | -.17 |
| 3-MSQ Wake | - | - | **1** | +.04 | +.02 | -.01 | +.04 | -.02 | -.07 | -.05 | -.05 | -.09 | +.11 | +.05 | **+.24*** | **+.29*** | **-.35*** | **-.36*** | **-.31*** |
| 4-WBT | - | - | - | **1** | **+.25*** | **-.57*** | **+.77*** | **+.60*** | **+.23*** | **-.32*** | **+.46*** | -.16 | -.03 | -.05 | +.09 | +.04 | +.07 | +.06 | +.05 |
| 5-WWT | - | - | - | - | **1** | **+.66*** | **+.81*** | +.17 | **+.44*** | **+.30*** | **+.37*** | **-.31*** | +.04 | +.002 | -.08 | +.14 | -.11 | -.09 | -.12 |
| 6-WTIB | - | - | - | - | - | **1** | +.09 | **-.32*** | +.20 | **+.50*** | -.05 | -.14 | +.05 | +.04 | -.14 | +.09 | -.15 | +.12 | -.15 |
| 7-WMPoS | - | - | - | - | - | - | **1** | **+.48*** | **+.43*** | +.006 | **+.52*** | **-.30*** | +.006 | -.03 | +.001 | +.11 | -.04 | -.02 | -.05 |
| 8-FBT | - | - | - | - | - | - | - | **1** | **+.49*** | **-.42*** | **+.85*** | **+.53*** | +.003 | +.002 | +.04 | +.04 | -.02 | +.02 | -.07 |
| 9-FWT | - | - | - | - | - | - | - | - | **1** | **+.58*** | **+.88*** | **+.61*** | +.04 | +.07 | -.07 | +.18 | -.12 | -.08 | -.15 |
| 10-FTIB | - | - | - | - | - | - | - | - | - | **1** | +.13 | +.14 | +.04 | +.07 | -.11 | +.15 | -.11 | -.11 | -.09 |
| 11-FMPoS | - | - | - | - | - | - | - | - | - | - | **1** | **+.66*** | +.03 | +.04 | -.02 | +.13 | -.09 | -.04 | -.13 |
| 12-SJL | - | - | - | - | - | - | - | - | - | - | - | **1** | +.03 | +.08 | -.03 | +.04 | -.07 | -.02 | -.10 |
| 13-PE | - | - | - | - | - | - | - | - | - | - | - | - | **1** | **+.29*** | **+.22*** | -.20 | -.17 | -.14 | -.18 |
| 14-PT | - | - | - | - | - | - | - | - | - | - | - | - | - | **1** | **+.25*** | -.08 | -.05 | -.04 | -.06 |
| 15-TP | - | - | - | - | - | - | - | - | - | - | - | - | - | - | **1** | **-.29*** | **-.25*** | **-.25*** | **-.22*** |
| 16-TE/B | - | - | - | - | - | - | - | - | - | - | - | - | - | - | - | **1** | -.19 | -.18 | -.18 |
| 17-PRMQ | - | - | - | - | - | - | - | - | - | - | - | - | - | - | - | - | **1** | **+.95*** | **+.95*** |
| 18-PRO | - | - | - | - | - | - | - | - | - | - | - | - | - | - | - | - | - | **1** | **+.80*** |
| 19-RETRO | - | - | - | - | - | - | - | - | - | - | - | - | - | - | - | - | - | - | **1** |

Note. rMEQ for reduced Morningness-Eveningness Questionnaire, MSQ Sleep for Mini-Sleep Questionnaire for Sleep factor, MSQ Wake for Mini-Sleep Questionnaire for Wake factor, WBT for Working Bed Time, WWT for Working Wake Time, WTIB for Working Time In Bed, WMPoS for Working Mid-Point of Sleep, FBT for Free Bed Time, FWT for Free Wake Time, FTIB for Free Time In Bed, FMPoS for Free Mid-Point of Sleep, SJL for Social JetLag, PE for Present Experience, PT for Past Time, TP for Time Pressure, TE/B for Time Expansion/Boredom, PRMQ for Prospective-Retrospective Memory Questionnaire for total score, PRO for Prospective Score and RETRO for Retrospective Score. In addition, °*p* = .01 and **p*≤ .001
